# Elucidating the Interactive Roles of Glia in Alzheimer's Disease Using Established and Newly Developed Experimental Models

**DOI:** 10.3389/fneur.2018.00797

**Published:** 2018-09-26

**Authors:** Heejung Chun, Ian Marriott, C. Justin Lee, Hansang Cho

**Affiliations:** ^1^Center for Glia-Neuron Interaction, Brain Science Institute, Korea Institute of Science and Technology, Seoul, South Korea; ^2^Department of Biological Sciences, University of North Carolina at Charlotte, Charlotte, NC, United States; ^3^Bio-Med, University of Science and Technology, Daejeon, South Korea; ^4^Department of Mechanical Engineering and Engineering Science, University of North Carolina at Charlotte, Charlotte, NC, United States; ^5^Center for Biomedical Engineering and Science, University of North Carolina at Charlotte, Charlotte, NC, United States; ^6^The Nanoscale Science Program, University of North Carolina at Charlotte, Charlotte, NC, United States

**Keywords:** neuroinflammation, Alzheimer's disease, astrogliosis, microgliosis, animal models, brain-on-a-chip, astrogliosis-microgliosis axis

## Abstract

Alzheimer's disease (AD) is an irreversible neurodegenerative illness and the exact etiology of the disease remains unknown. It is characterized by long preclinical and prodromal phases with pathological features including an accumulation of amyloid-beta (Aβ) peptides into extracellular Aβ plaques in the brain parenchyma and the formation of intracellular neurofibrillary tangles (NFTs) within neurons as a result of abnormal phosphorylation of microtubule-associated tau proteins. In addition, prominent activation of innate immune cells is also observed and/or followed by marked neuroinflammation. While such neuroinflammatory responses may function in a neuroprotective manner by clearing neurotoxic factors, they can also be neurotoxic by contributing to neurodegeneration *via* elevated levels of proinflammatory mediators and oxidative stress, and altered levels of neurotransmitters, that underlie pathological symptoms including synaptic and cognitive impairment, neuronal death, reduced memory, and neocortex and hippocampus malfunctions. Glial cells, particularly activated microglia and reactive astrocytes, appear to play critical and interactive roles in such dichotomous responses. Accumulating evidences clearly point to their critical involvement in the prevention, initiation, and progression, of neurodegenerative diseases, including AD. Here, we review recent findings on the roles of astrocyte-microglial interactions in neurodegeneration in the context of AD and discuss newly developed *in vitro* and *in vivo* experimental models that will enable more detailed analysis of glial interplay. An increased understanding of the roles of glia and the development of new exploratory tools are likely to be crucial for the development of new interventions for early stage AD prevention and cures.

## Introduction

### Neurotoxic glial activation exacerbates AD dementia

Many researchers have genetically modified human AD genes in mice and rats to overexpress Aβ peptides and/or tau proteins to mimic Aβ plaques and/or NFTs, which are features of human AD brain pathology. These animal models have, therefore, been widely used to test potential AD therapies, but more than 20 agents that have shown promise in these models have failed in clinical trials (1, 2), raising suggestions that amyloid and tau may need to be targeted decades before clinical symptoms appear, and causing some to even question the validity of the amyloid and tau hypothesis. In addition to Aβ plaque and NFT deposition, it has been recently recognized that brain inflammation involving glial cell activation is a prominent feature of AD. For example, increased inflammatory mediator expression has been reported in postmortem brains of AD patients (3, 4), and epidemiological studies have linked the use of anti-inflammatory drugs with a reduced risk for this disorder (5, 6). It is known that the degree of glial cell activation and their interplay correlates with the extent of brain atrophy and cognitive impairment (7, 8). It is, therefore, reasonable to suggest that glial neuroinflammatory responses, and those of microglia and astrocyte in particular, exacerbate the neurodegeneration associated with AD. In this review, we will discuss the available evidence that supports such a hypothesis.

### Reactive astrocytes and activated microglia in neuroinflammation

Astrocytes are the most abundant glial cells in the brain and are responsible for brain homeostasis. In pathological conditions, reactive astrocytes are ubiquitously detected throughout the central nervous system (CNS). Reactive astrocytes are identified by increased expression of intermediate filament proteins such as glial fibrillary acidic protein (GFAP) and vimentin. The astrocyte reactivity can be categorized as mild/moderate or severe. In brain injury model, the mild/moderate reactive astrocytes show hypertrophy having ramified processes without proliferation, whereas severe reactive astrocytes have proliferating potential with severe hypertrophy (9). Recently, Liddelow et al. categorized reactive astrocytes into A1 and A2 in conditions of lipopolysaccharide (LPS) treatment and middle cerebral artery occlusion (MCAO), and characterized these astrocytes as neurotoxic or neuroprotective, respectively. They identified that the A1 astrocytes were triggered by activated microglia through secreting tumor necrosis factor (TNF), interleukin-1α (IL-1α) and complement component 1q (C1q) and lost many neuroprotective functions of astrocytes (10).

**Figure 1:**
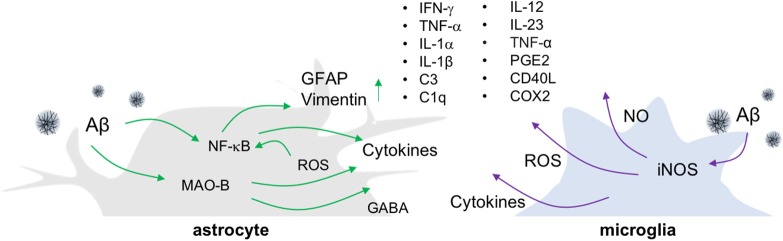
Release of inflammatory molecules from activated glial cells in AD. In an Aβ-overproducing animal model of AD, inflammatory molecules such as cytokines, ROS/RNS and gliotransmitters are released from reactive astrocytes and activated microglia.

Microglia, resident myeloid cells in a CNS, continually survey their microenvironments in normal and diseased brains while providing immune surveillance and activation in response to infection, non-infectious diseases, and injury (11–13). Although microglial hyper-activation or dysfunction is a potential mechanism leading to neurodegenerative and neuroinflammatory diseases, the roles of microglia are still under debate (14). There have also been attempts to understand the heterogeneity of activated microglia, as M1 and M2. M1 microglia are classically activated microglia, which produce inflammatory cytokines and reactive oxygen species (ROS) (Figure 1), whereas M2 microglia are in a state of alternative activation that show an anti-inflammatory phenotype (15). However, these categorizations remain under consideration because microglial activation shows more complex variations in phenotypes (16).

### Neuroinflammatory aspects of animal models of AD and their limitations

The most widely employed transgenic animal models for AD display substantial reactive gliosis that includes activated astrocytes and microglia (17, 18). These cellular responses are detected before the appearance of Aβ plaques and NFT pathology (19, 20). P301S tau transgenic mice are a model for tauopathy and exhibit not only aggregated tau, but also the production of inflammatory cytokines including IL-1β and glial activation around tau-positive neuronal cells (21). Such microglial activation precedes NFT formation and appears in 3-months old P301S tau transgenic animals (22). Similarly, in the APP/PS1 mice model, the reactive astrocytes near Aβ plaques look increasingly hypertrophied as AD develops. In addition to glial activation, these mice show enhanced levels of gliotransmitters such as gamma-aminobutyric acid (GABA) (23), more ROS, elevated production of cytokines including tumor necrosis factor-alpha (TNF-α), interferon-gamma (IFN-γ), IL-1β, IL-1α, chemoattractant protein-1, and greater expression of the inflammatory mediators cyclooxygenase-2 (COX-2) and C1q (17, 24) (Figure 1). Importantly, the degree of inflammatory cell activation and cytokine production correlates with disease progression and severity in mouse models of AD (23, 25).

To investigate the causal relationship between such neuroinflammatory responses and AD pathology, genetic and pharmacological manipulation of inflammatory components including IL-12, IL-23, TNF-α, prostaglandin E2, and cluster of differentiation (CD) 40 ligand (CD40L) has been performed in animal models of AD. In these studies, inflammatory factor inhibition has been found to decrease Aβ plaque loads (26–28). However, the role of neuroinflammation in other aspects of AD pathology, such as neuronal death or cognitive decline, remains elusive. Additionally, most animal models of AD that show amyloidogenesis fail to exhibit the tauopathy, brain atrophy, and neuronal death, that are common features in AD patients (29, 30). To overcome these limitations, researchers have made triple transgenic mice (3XTg-AD) harboring a tau transgene (tau_P301L_) in addition to APP_SWE_ and presenilin-1 (PS1_M146V_) mutations (31), or developed promoters exclusively expressing transgenes in neurons. Some of these mice show neuronal death and brain atrophy in specific brain regions while showing progressive Aβ plaque and NFT formation (32, 33). However, such tau transgenic mice have limitations for the study of AD due to the various isoforms of the tau gene that are possible. The tau gene generates six isoforms by alternative splicing, which are divided depending on the combination of exon 2/3 and exon 10. Tau transgenic mice that overexpress the longest form of the tau gene and/or modulate tau phosphorylation through glycogen synthase kinase 3 beta (GSK-3β) paradoxically do not exhibit NFT.

## The astrogliosis-microgliosis axis (AMA) contributes to AD progression

**Figure 2:**
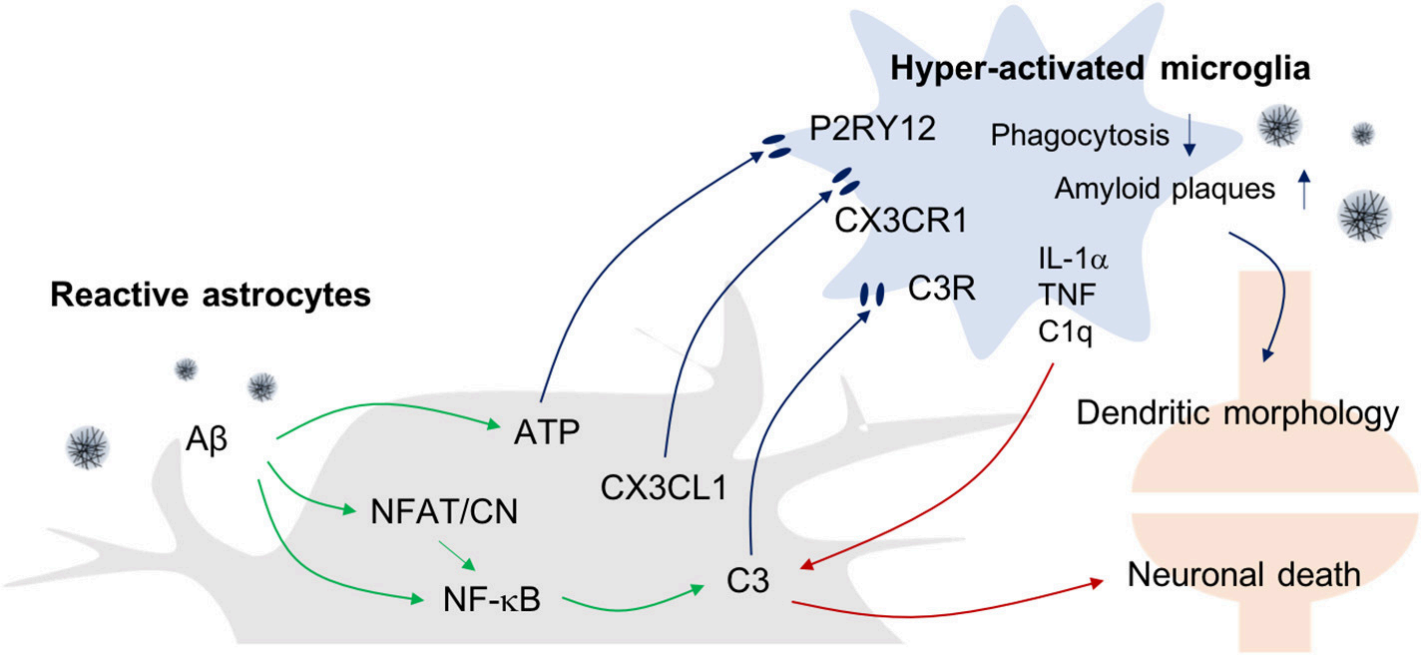
Astrogliosis-Microgliosis Axis (AMA) in AD. Schematic showing the crosstalk between reactive astrocytes and hyper-activated microglia in AD. It has been reported that reactive astrocytes release ATP, CX3CL1, and C3 to activate microglia, whereas the activated microglia release inflammatory molecules such as IL-1α, TNF-α, and C1q to increase C3 expression in astrocytes, which consequently causes an increase in AD pathology indices such as Aβ plaques and neuronal death.

Functional studies of astrogliosis and microgliosis and their relationship with AD have been performed in various experimental conditions. However, the relationship between gliosis in AD pathogenesis is complex and remains unclear despite the fact that resolving the timeline of AD pathology is essential to defining the cellular and molecular mechanisms underlying AD pathogenesis. For example, an increase in Aβ levels and astrocyte activation are found to occur early, even before the mild cognitive impairment (MCI) phase and reach a plateau when clinical symptoms appear (3, 34). In contrast, microglial activation, tau pathology, and neuronal death occur later in the disease and correlate with the severity of clinical symptoms (3). Below, we summarize the available evidence for astrocyte and microglia crosstalk during AD pathogenesis (Figure 2).

### From astrogliosis to microgliosis

Under physiological conditions, astrocytes modulate the status of microglial activation. In the presence of astrocytes, Aβ toxin-induced microglial responses such as reactive morphological changes, inducible nitric oxide synthase (iNOS) induction, and decreased reductive metabolism, are attenuated (35). Astrocyte-derived transforming growth factor (TGF)-β1 deactivates microglial cells and abolishes neurotoxicity (36) and its modulatory effect involves the activation of the Smad3 pathway, which is down regulated in AD patients (37), and the activation of mitogen-activated protein kinase (MAPK)- extracellular-signal-regulated kinase (ERK) pathways (38) that also appear to be neuro-protective (39). Dynamic regulation of Smad, phosphoinositide 3-kinase (PI3K), and MAPK pathways, which are associated with TGF-activity in addition to inflammatory cytokine-mediated effects, is an important component in the control of cell integrity and inflammatory responses. The abolition or decreases in the level of Smad3, a major effector pathway for anti-inflammatory responses, would therefore modify the regulatory feedback signals that result from inflammation.

However, reactive astrocytes under pathological conditions elicit microglial activation *via* several mechanisms that subsequently lead to AD pathology. When the reactivity of astrocytes is attenuated by blocking the inflammatory calcineurin/nuclear factor of activated T-cells (NFAT) signaling pathway in the presence of Aβ, microglial activation is significantly reduced, indicating that reactive astrocytes utilize that pathway to direct microglial activation. Moreover, such reactive astrocyte attenuation and diminished numbers of activated microglia was associated with reduced amyloid levels and improved cognitive and synaptic functions in APP/PS1 mice (40). These findings therefore suggest that microglial activation underlies the deleterious effects of reactive astrocytes in AD progression.

With regard to the possible mechanisms linking astrogliosis to microglial activation, it has been reported that C3 released from Aβ-treated astrocytes can upregulate C3a receptor (C3aR) expression by microglia (41). Importantly, nuclear factor of kappa-light-chain-enhancer of activated B cells (NF-κB) hyperactivation due to inhibitor of κB kinase (IKK) knockout (KO) results in complement expression by astrocytes because C3 protein secretion is driven by NF-κB activation in these cells, and such responses worsen Aβ-associated pathology, with reduced numbers of synapses and shortened dendritic lengths, and impaired synaptic functions, due to reduced microglial Aβ phagocytosis in AD mouse models (42, 43). Conversely, C3aR antagonists reduce Aβ plaques formation and attenuate microgliosis. Together, these data suggest that astrocytic activation in response to Aβ leads to microgliosis *via* the C3-C3aR pathway.

In addition, reactive astrocyte-mediated increases in the levels of synaptically localized C1q may be responsible for the microglial activation that results in age-dependent cognitive dysfunctions (14). In support of this idea, Bialas et al. showed that severe neuroinflammation might be a cause of autoimmune diseases such as lupus (44). Using live-cell imaging approaches in a mouse model of interferon over-expression, hyperactivated microglia were seen to ingest synaptic debris from neurons resulting in a reduced synaptic network.

In addition to complement components, other evidence has shown that extracellular ATP released from astrocytes can activate microglia *via* purinergic receptors. ATP is known to be released by N-methyl-D-aspartate (NMDA)-sensitive neurons (45), damaged astrocytes, and leaky blood vessels (46). ATP recruits and activates microglia to sites of injury *via* the P2RY12 purinergic receptor leading to synapse remodeling (47). Finally, astrocytes can also express the chemokine C-X3-C motif chemokine ligand 1 (CX3CL1) in inflammatory conditions and microglia express its receptor, C-X3-C motif chemokine receptor 1 (CX3CR1). Interestingly, it has been reported that CX3CR1-deficiency increases the functional connectivity of neural circuits while decreasing the number of microglia. However, the mechanism underlying this effect remains unclear (48, 49).

### From microgliosis to astrogliosis

Recently, there have been attempts to categorize and characterize reactive astrocytes into distinct A1 and A2 phenotypes in the brain. It is suggested that the A1 phenotype represents reactive astrocytes that are induced by systemic LPS injection and whose function is detrimental to neurons, whereas A2 reactive astrocytes act in a protective manner and are induced in conditions such as the MCAO stroke model (10). Interestingly, in this study, the formation of A1 reactive astrocytes appeared to be dependent on the activation of microglia as colony stimulating factor 1 receptor (Csf1r) KO mice that lack this cell type fail to form this astrocytic phenotype following LPS challenge. This led these investigators to suggest that detrimental A1-type reactive astrocytes are induced secondary to the release of proinflammatory factors such as IL-1α, TNF-α, and C1q by microglia. Interestingly, C3 has been used as a marker for A1-type reactive astrocytes, and this complement component is ubiquitously expressed in AD brain astrocytes. However, it remains to be seen whether the microglia-induced detrimental reactive astrocytes seen following systemic LPS-treatment model are replicated in AD models.

## Established and new experimental models to study the role of gliosis in AD

Appropriate and controllable models are required if we are to determine the role of reactive astrocytes and activated microglia in neurodegenerative diseases such as AD, and define the interplay between these cell types. Below, we describe currently employed models to evaluate and manipulate astrocyte and microglial functions, and discuss newly developed models that may prove useful in establishing the role of gliosis in AD.

### Experimental animal models to modulate astrocyte reactivity in AD

#### MAO-B-modulating reactive astrocyte models

Monoamine oxidase B (MAO-B) has been implicated in the pathogenesis of AD due to the increased expression of this molecule in astrocytes in the brains of AD patients (50) and adjacent to Aβ plaques in animal model of AD (23). The activation of MAO-B results in the aberrant production and release of GABA by reactive astrocytes, leading to reduced spike probability of granule cells *via* presynaptic GABA receptors (23). In addition, MAO-B produces hydrogen peroxide, a type of ROS, further contributing to AD pathology. As such, a reduction in ROS-induced oxidative stress *via* MAO-B activity inhibition would be expected to delay the progression of the disease. For these reasons, MAO-B inhibitors have been used to pharmacologically block astrogliosis in experimental models of AD. The MAO-B inhibitor selegiline has been reported to reduce the reactivity of astrocytes in APP/PS1 mice and this effect is associated with reductions in memory impairment (23). Interestingly, transgenic animals that conditionally overexpress MAO-B in GFAP-positive astrocytes demonstrate elevated astrocyte reactivity that is associated with increased ROS formation and neuronal loss, a phenotype that is reversed by treatment with the MAO-B inhibitor sembragiline (51). Clearly, further study of the therapeutic effects of MAO-B inhibitors on astrogliosis in AD patients is warranted.

#### Virus-mediated reactive astrocytes models: HSV, AAV, and adenovirus

Viruses have been implicated in the etiology of AD through the induction of acute and chronic diseases in the CNS (52, 53). Recently, Eimer et al. suggested that herpes simplex virus-1 (HSV-1) infection aggravates Aβ deposition and AD progression by protective amyloidosis (54). Reactive astrocytes have been suggested to be one of the key cellular mediators of virus-induced CNS pathology. In the CNS, it has been reported that HSV-1 can induce astrocyte activation (55) and these cells have been shown to take up Zika virus *via* the astrocytic protein AXL (56). Recently, it was reported that HSV-1 causes the activation of GSK-3, which subsequently phosphorylates amyloid precursor protein (APP) (57). However, the mechanisms underlying virus-induced reactive astrogliosis are still unclear and the high degree of association between such infections and AD indicate that virus-mediated experimental models are required to further investigate this link.

Previously, AAV virus has been used to induce reactive astrocytes *in vivo* and the AAV2/5-gfp104-eGFP virus has been shown to increase the number of reactive astrocytes in a titer-dependent manner (58). In this model, authors found that reactive astrocytes downregulate the expression level of glutamate synthetase, impair inhibitory neurotransmission, and affect network hyperexcitability. Similarly, Woo et al. showed that the adenovirus, Adeno-GFAP-GFP, induces astrocyte reactivity in the dentate gyrus region of the mouse hippocampus (59), with astrocytes in this region becoming hypertrophied following virus-injection compared to astrocytes in uninfected mice. These models could, therefore, be readily employed to induce focal reactive astrogliosis in specific brain regions of interest. However, it must be noted that the use of such models will require caution to distinguish the effects of reactive astrogliosis from virally-induced inflammatory responses.

#### GFAP- and vimentin-modulated reactive astrocyte AD models

Because astrocyte reactivity is characterized by hypertrophied processes and increased expression of intermediate filaments such as GFAP and vimentin, the role of astrocyte intermediate filaments in AD has been investigated by crossing mice genetically deficient in GFAP and vimentin with APP transgenic mice. While the validity of these models remains controversial, studies using these models has shown that GFAP and vimentin KO in APP/PS1 mice is associated with an almost two-fold increase in Aβ plaque formation at 8 and 12-months of age. In these triple transgenic mice, APP processing and soluble and interstitial fluid Aβ levels were unchanged, which suggests that Aβ degradation, rather than Aβ generation, is affected by the deletion of astrocyte intermediate filaments (IF). Astrocytes in GFAP and vimentin deficient animals showed marked alterations in their morphology near Aβ plaques, with little process hypertrophy and lacking contact with adjacent Aβ plaques. Moreover, these mice showed a marked increase of neurite dystrophy. Such findings indicate that activation-associated changes in astrocyte morphology limit Aβ plaque growth and attenuate plaque-related dystrophic neurites (60, 61). However, caution is needed when interpreting these studies, as intermediate filament KO appears to result in additional effects that include the absence of endothelin B receptor protein expression increases in GFAP/vimentin KO mice that normally occur in reactive astrocytes (62).

#### Animal models that feature the modulation of inflammatory signaling pathways in reactive astrocytes

Astrocyte reactivity can be blocked by genetic manipulation using AAV-GFAP-VIVIT in which VIVIT is a synthetic peptide that disrupts the physical interaction between calcineurin and NFAT. It interferes with NFAT activation in astrocytes and consequently reduces cytokine release and neuroinflammation. *In vivo* application of this tool to APP/PS1 mice significantly reduces AD pathology, reducing amyloid levels and improving cognitive functions, indicating that reactive astrocytes act in a deleterious manner (40). This is supported by *in vitro* studies that also showed astrocyte VIVIT expression ameliorates the neurotoxic effects of activated astrocytes on neighboring neurons (63, 64). Another strategy to block astrocyte reactivity is to knockout/knockdown IKKβ expression or overexpress its dominant negative form in these cells (65–67). Such approaches inhibit the inflammatory NF-κB pathway in reactive astrocytes. Finally, deletion of aquaporin 4 (AQP4) has also been employed to block astrocytic functions, and deletion of this channel has been shown to exacerbate brain Aβ accumulation and memory deficits in APP/PS1 mice (68). However, it is unclear from the limited number of studies employing these models whether and how the reactivity of reactive astrocytes contributes to AD pathogenesis.

### Experimental animal models to modulate microglia activation in AD

Microglial hyper-activation is believed to be neurotoxic and, therefore, blocking microglial activation is predicted to attenuate disease progression. Pharmacological and genetic manipulation of microglial activation in animal models of AD have been performed to determine the contribution of microglia activation to AD pathogenesis. Asraf et al. reported that blocking microglial activation through Captopril, which inhibits angiotensin-converting enzyme (ACE) and blocks the formation of angiotensin II (Ang II), decreased LPS-induced NO release and regulated iNOS, TNF-α, and IL-10 in BV2 microglia cells. This tool was also applied to an *in vivo* AD mouse model, 5X FAD. Intranasal treatment with Captopril for 2-months ameliorated microglial activation and decreased Aβ burden, indicating that microglial activation exacerbates AD pathology (69). On the other hand, Manocha et al. reported that inhibition or deletion of NFAT 2c isoform, which is the most highly expressed gene in microglial culture, modulated microglial activation and blocked the release of cytokines from microglia. When NFATc2 KO mice were crossed with APP/PS1 mice, cytokine levels and microgliosis were reduced. However, there was no effect on plaque load (70). On the other hand, phagocytic roles of microglial triggering receptor expressed on myeloid cells 2 (TREM2) have been reported in AD and in mice. TREM2 deficiency results in a reduced microglial inflammatory response (71). By crossing TREM2 deficient mice crossed with AD mouse models, researchers have shown that TREM2-deficient microglia are ineffective at either clustering around and/or removing fibrillary Aβ (72, 73). These studies suggest that microglial activation exacerbates AD pathogenesis.

### Novel *in vitro* culture models of human glia in AD

#### A microfluidic chip to monitor microglial responses to disease-related soluble cues

*In vivo* studies of microglial migration in AD have been hampered by the complexity of the effects of Aβ on microglia due to the multiple forms of Aβ and heterogeneous microglial activation (74). Aβ peptides form deposits of insoluble forms of this molecule surrounded by a mixture of soluble oligomeric Aβ (75), accumulated microglia, and dystrophic neurites (76). Activated microglia can take on a variety of morphologies that include rounded, ramified, rod-like, and amoeboid forms that are followed by motile activation, and these changes complicate visual tracking of individual cells. Previous microfluidic attempts to study rat microglial migration in the presence of short-lived damaged axons (77) did not establish long-lasting chemoattractant gradients, and failed to conclusively differentiate gradual microglial accumulation from heterogeneous activation and random navigation (78, 79). More recently, we have developed a novel microfluidic chemotaxis platform to study the regulated and stimuli-selective microglial motility (80). To understand the specific role of Aβ in microglial accumulation, we generated soluble Aβ (sAβ) gradients that lasted a week, patterned insoluble surface-bound Aβ (bAβ) to mimic the Aβ signature in AD brains, and were able to isolate human microglia responding to Aβ in this platform. We evaluated the Aβ sensitivity of primary human microglia isolated from human fetal brain (HMG 030, Clonexpress, Inc.) and adult human microglial cell lines (T0251, ABM Inc.) and characterized their responses. In addition, we were able to monitor single cell changes in microglial morphology in real time in response to soluble Aβ that were associated with directional migration. We found that soluble monomeric and oligomeric Aβ can act as a microglial chemoattractant at a broad range of concentrations from picomolar to nanomolar that correspond to levels in normal and AD brains, respectively. Importantly, we were also able to discern co-localization of microglia to insoluble Aβ in this model that was similar to Aβ plaques seen in AD.

#### A microfluidic model to assess crosstalk between central and peripheral immunity in AD

The accumulation of immune cells in the brain parenchyma is a critical step in the progression of neuroinflammatory diseases including AD. While the mechanisms underlying central immunity activation and Aβ clearance are well studied in the context of AD pathogenesis, the mechanisms responsible for the recruitment of peripheral immune cells from the blood stream to CNS disease sites are less clear. Peripheral immune cells including T and B lymphocytes, monocytes, and neutrophils, have been identified in the brains of human AD patients and corresponding animal models (81–83). Among these immune cells, neutrophils are of great interest because they are key effector cells in many inflammatory responses, and they show a remarkable ability to migrate within and through blood vessels (84, 85). To better assess the potential for neutrophil recruitment and activation in AD, we have reconstituted an AD microenvironment in a microfluidic model that includes the induction of cytokine/chemokine production by human microglial cells stimulated with Aβ, and employed this model to investigate the recruitment of human neutrophil in the context of innate-peripheral immunity crosstalk (86). In this model, we observed that Aβ stimulated microglial cells induce the robust recruitment of human neutrophils concomitant with the release of mediators including IL-6, IL-8, chemokine ligand (CCL) 2, CCL3/4, and CCL5. We subsequently confirmed a role for IL-6, IL-8, and CCL2 in neutrophil recruitment with the demonstration that such responses were attenuated by the presence of neutralizing antibodies against these factors. Interestingly, the recruited neutrophils in this system prompted the release of additional inflammatory mediators such as macrophage migration inhibitory factor (MIF) and IL-2. As such, this microfluidic system shows great promise for the study of chemotactic crosstalk between resident CNS cells and circulating leukocytes in AD and may prove useful in determining the therapeutic potential of targeting neutrophil neuroinflammatory activity to limit AD pathogenesis.

#### *In vitro* generation of iPSC-derived microglia-like cell

Haenseler et al. developed microglia-like cells by co-culturing iPSC-derived macrophages with iPSC-derived cortical neurons and demonstrated the expression of major microglia-specific markers and neurodegenerative disease-relevant genes (87). In transcriptome analyses, the microglia isolated using CD11β show upregulation of the six key microglia-specific genes: MER proto-oncogene, tyrosine kinase (MERTK), G-protein coupled receptor 34 (GPR34), protein S (PROS1), C1QA, growth arrest-specific 6 (GAS6), and P2RY12 in relevant homeostatic pathways and downregulation of antimicrobial pathways. Also, microglia expressed neurodegeneration-involving genes: fermitin family member 2 (FERMT2), TREM2, apolipoprotein E (APOE), and ubiquitin C-terminal hydrolase L1 (UCHL1), AD genes: APP, phosphatidylinositol binding clathrin assembly protein (PICALM), and CD33, PD genes: Parkinson disease 15 (PARK15), PTEN-induced putative kinase 1 (PINK1), synuclein alpha (SNCA), and protein deglycase (DJ-1), motor neurone disease (MND) genes: C9orf72, TAR DNA-binding protein (TARDBP), and superoxide dismutase 1 (SOD1). Co-cultured microglia showed various alterations including changes in microglia-related protein expression and morphogenesis, and elevated motility and phagocytosis.

Further to this, Abud et al. generated human iPSC-derived microglia-like cells (iMGLs) with a two-step culturing protocol. Whole-transcriptome analysis demonstrated high similarity to cultured adult and fetal human microglia by expressing microglial genes: P2RY12, GPR34, C1Q, Cdk5, and Abl enzyme substrate 1 (CABLES1), basic helix-loop-helix family, member e41 (BHLHE41), TREM2, PROS1, APOE, solute carrier organic anion transporter family member 2B1 (SLCO2B1), solute carrier family 7 member 8 (SLC7A8), peroxisome proliferator activated receptor delta (PPARD), and crystallin beta B1 (CRYBB1) (88). Functional assessments revealed that iMGLs secrete eight different cytokines and chemokines, including TNF-α, CCL2, CCL4, and CXCL10 in response to IFN-γ or IL-1β, migrate along an ADP gradient, produce calcium transients initiated via P2RY12, and exhibit robust C1q/CR3-mediated phagocytosis. In particular, they showed that iMGLs can internalize fluorescently labeled fibrillar Aβ and tau oligomers. In addition to genes involved in AD pathology, iMGLs express other neurodegenerative disease-relevant genes, including APP, PSEN1/2, huntingtin (HTT), progranulin (GRN), TARDBP, leucine-rich repeat kinase 2 (LRRK2), C9orf72, SOD1, valosin-containing protein (VCP), and FUS, which are correlated with amyotrophic lateral sclerosis (ALS), Huntington's disease (HD), frontal temporal dementia (FTD), and dementia with Lewy bodies (DLB), which supports the potential for these cells in the study of a variety of neurological diseases.

#### A 3D organotypic model of the human AD brain

We have recently developed a new 3D organotypic human cell AD brain model by tri-culturing human AD neurons, astrocytes, and adult microglial cells in a 3D microfluidic platform (3D hNeuroGliAD) (89). This model replicates key characteristic features of AD including accumulation of Aβ, phosphorylated tau (pTau) accumulation, and AD neuron and astrocyte damage associated with microglial inflammatory responses. A central chamber was loaded with immortalized AD human neural progenitor cells (hNPCs), or iPSC-derived human AD NPCs provided by Drs. Tanzi and Kim (90), suspended in a growth factor-reduced Matrigel 150 μm in height, and differentiated into human AD neurons and astrocytes on the chip, while an angular chamber was loaded with human adult microglia. The central and angular chambers were linked by migration channels that formed soluble factor gradients from the central chamber and served as mechanical barriers to spontaneously activated microglia.

Our model provided representative AD signatures that included pathological accumulation of Aβ and pTau, NFT-like structure formations inside neurons, and IFN-γ production by astrocytes. Microglial morphological changes and migration toward the central chamber began at 48 h following cell seeding with the microglial cells elongating as the length of their somata (cell bodies) increased disproportionally. Furthermore, microglia exposed to soluble AD cues from the cultured AD neurons and astrocytes showed an up-regulation in the expression of activation markers including CD11b. This 3D AD brain model was associated with significant increases in the release of several key chemokines including CCL2 (2.1-fold), CCL5 (26-fold), CXCL10 (2.6-fold), and CXCL12 (1.2-fold), and inflammatory cytokines such as IL-6 (2.2-fold), IL-8 (2.7-fold), and TNF-α (1.3-fold), by microglia as compared to controls. We also observed the unique production of the leukocyte growth factors, granulocyte-macrophage colony-stimulating factor (GM-CSF) and granulocyte colony-stimulating factor (G-CSF) in our AD model that was in contrast to the expression of anti-inflammatory markers including IL-1RA, IL-10, and TGF-β, that was very low or undetectable.

**Figure 3:**
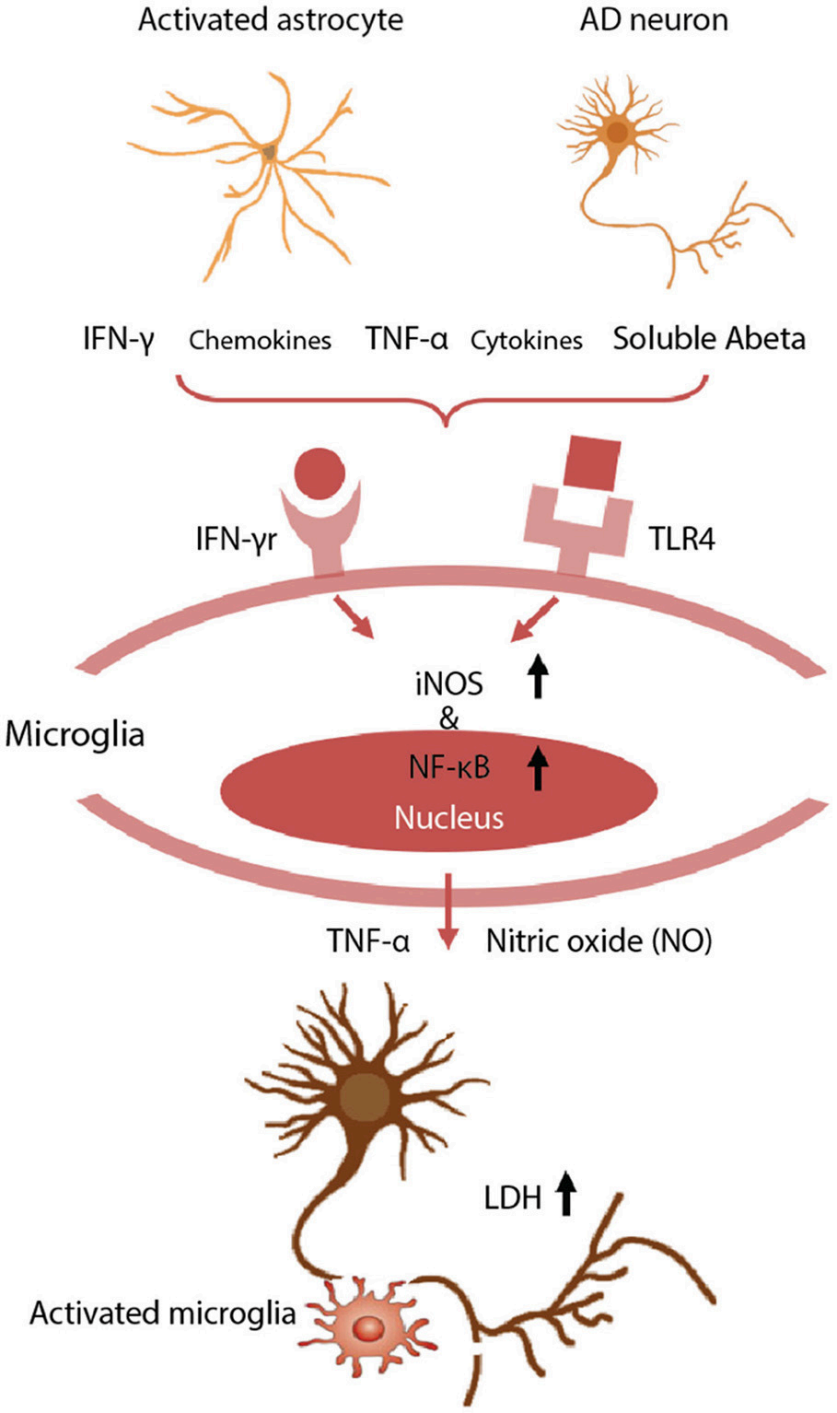
Neurotoxic glia interactions mediated by TLR4 and IFN-γ receptor. Schematic of detrimental astrocyte-microglia activation in a 3D human AD model. The combination of Aβ derived from AD neurons and inflammatory cytokines from reactive astrocytes are detected by microglia *via* TLR4 and IFN-γ receptors. Then, the microglia subsequently activate the expression of iNOS and NF-κB, and release TNF-α and cytotoxic NO, leading to neurodegeneration. Neuronal damage was assessed using a LDH assay to quantify membrane damage. Reproduced/adapted from reference [(89)**]**.

We have observed significant neuronal loss in this human tri-culture 3D model at 9 weeks that corresponds to a late AD stage (Figure 3) (89). The mechanisms underlying such neuronal damage in our 3D AD brain model were investigated by examining the astrogliosis-microgliosis axis using glial cell monocultures. We found that Aβ derived from AD neurons and IFN-γ produced by reactive astrocytes combined to activate microglia *via* toll like receptor 4 (TLR4) and IFN-γ receptors, respectively, and these cells subsequently expressed iNOS, and released TNF-α and cytotoxic nitric oxide (NO), leading to neuronal damage. In contrast, exposure to IFN-γ alone did not trigger TNF-α or NO release by microglia. NO levels were increased by 9.1-fold in 3D AD brain models cocultured with microglia, but this mediator was not detectable in the absence of microglia or models of early stage AD due to a lack of IFN-γ production. Blocking microglial TLR4 receptors, either with a neutralizing anti-TLR4 antibody or a TLR4 antagonist, decreased the levels of TNF-α, iNOS, and NO release, indicating that enhanced iNOS expression and concomitant NO release is mediated by a TLR4 dependent mechanism. Interestingly, the release of lactate dehydrogenase (LDH), a biochemical marker of cell death, was significantly decreased in the presence of TLR4 antagonist, LPS-RS, in our 3D AD brain model. These data indicate that microglia in a 3D AD brain model induce neuronal loss through an IFN-γ and Aβ dependent mechanism, which could have critical implications for future AD therapy drug screening efforts (87).

## Future prospects

Recent clinical trial failures of AD drugs that target Aβ have led researchers to consider alternative molecular targets that involve neuroinflammation, in particular, the activation of microglia and astrocytes. A major function of astrocytes and microglia appears to be the degradation of toxic molecules such as Aβ and hyperphosphorylated tau. However, it is unclear how this degradative function results in reactivated or hyper-activated astrocytes and microglia. While the appearance of reactive astrocytes and activated microglia precedes the symptomatic and neurodegenerative stages of AD, it is still unclear whether astrogliosis precedes microgliosis or vice versa. Because microglia are far more mobile than astrocytes, it is possible that astrocytes that are normally in contact with neurons, especially at the synaptic junctions, sense danger and alert nearby microglia to sites of injury and/or amyloid deposits. Therefore, reactive astrocytes might be the initial trigger for the cascade of events that leads to neuroinflammation and neurodegeneration. The alternative is that microglia, whose principle function is immune surveillance, are the first to detect danger and instruct nearby astrocytes, *via* released cytokines and other inflammatory mediators, to recruit more microglia in a feed-forward manner that leads to neuroinflammation and neurodegeneration. This question is extremely difficult to address with current *in vivo* and *in vitro* experimental models due to a number of limitations that we have discussed. However, some of the newly developed reactive astrocyte models may prove useful in addressing such questions. Furthermore, our newly developed multicellular human AD *in vitro* models have great potential as tools to define the molecular and cellular mechanisms underlying reactive astrogliosis and microglial hyper-activation and their role in AD pathogenesis. For example, recently identified reactive astrocyte markers, such as MAO-B, and the microglial marker, TREM2, could be investigated at the molecular and cellular level using these recently developed *in vivo* and *in vitro* AD models (91). While glial responses might be intended to be protective for individual cells and/or the brain tissue as a whole, the accumulated cellular defensive responses could result in the chronic brain alterations associated with AD. As such, defining the cellular mechanisms underlying these responses could help to understand the controversial roles of glial activation during the progression of AD pathology. Moreover, an examination of the events responsible for astrogliosis and microgliosis will provide mechanistic insights into disease progression, and may provide novel diagnostic markers or even therapeutic interventions at the very earliest stages of AD. Considering the recent discoveries implicating astrogliosis and microgliosis in AD pathology, and an array of newly developed models to pursue such avenues of investigation, there is reason for optimism for the treatment of AD.

## Author contributions

HeC, IM, CL, and HaC reviewed literature, outlined, wrote the manuscript, and prepared figures. All authors read and edited the manuscript extensively.

### Conflict of interest statement

The authors declare that the research was conducted in the absence of any commercial or financial relationships that could be construed as a potential conflict of interest.

## References

[1] Doody RS Thomas RG Farlow M Iwatsubo T Vellas B Joffe S. Phase 3 trials of solanezumab for mild-to-moderate Alzheimer's disease. N Engl J Med. (2014) 370:311–21. 10.1056/NEJMoa1312889 24450890

[2] Hawkes N. Merck ends trial of potential Alzheimer's drug verubecestat. BMJ (2017) 356:j845. 10.1136/bmj.j845 28202490

[3] Jack CR Jr Knopman DS Jagust WJ Shaw LM Aisen PS Weiner MW. Hypothetical model of dynamic biomarkers of the Alzheimer's pathological cascade. Lancet Neurol. (2010) 9:119–28. 10.1016/S1474-4422(09)70299-6 20083042 PMC2819840

[4] Morimoto K Horio J Satoh H Sue L Beach T Arita S. Expression profiles of cytokines in the brains of Alzheimer's disease (AD) patients compared to the brains of non-demented patients with and without increasing AD pathology. J Alzheimers Dis. (2011) 25:59–76. 10.3233/JAD-2011-101815 21368376 PMC3572941

[5] Lichtenstein MP Carriba P Masgrau R Pujol A Galea E. Staging anti-inflammatory therapy in Alzheimer's disease. Front Aging Neurosci. (2010) 2:142. 10.3389/fnagi.2010.00142 21152343 PMC2998033

[6] Szekely CA Zandi PP. Non-steroidal anti-inflammatory drugs and Alzheimer's disease: the epidemiological evidence. CNS Neurol Disord Drug Targets (2010) 9:132–9. 10.2174/187152710791012026 20205647

[7] Cagnin A Brooks DJ Kennedy AM Gunn RN Myers R Turkheimer FE. *In-vivo* measurement of activated microglia in dementia. Lancet (2001) 358:461–7. 10.1016/S0140-6736(01)05625-2 11513911

[8] Parachikova A Agadjanyan MG Cribbs DH Blurton-Jones M Perreau V Rogers J. Inflammatory changes parallel the early stages of Alzheimer disease. Neurobiol Aging (2007) 28:1821–33. 10.1016/j.neurobiolaging.2006.08.014 17052803 PMC2198930

[9] Sofroniew MV. Molecular dissection of reactive astrogliosis and glial scar formation. Trends Neurosci. (2009) 32:638–47. 10.1016/j.tins.2009.08.002 19782411 PMC2787735

[10] Liddelow SA Guttenplan KA Clarke LE Bennett FC Bohlen CJ Schirmer L. Neurotoxic reactive astrocytes are induced by activated microglia. Nature (2017) 541:481–7. 10.1038/nature21029 28099414 PMC5404890

[11] Graeber MB. Changing face of microglia. Science (2010) 330:783–8. 10.1126/science.1190929 21051630

[12] Kettenmann H Hanisch UK Noda M Verkhratsky A. Physiology of microglia. Physiol Rev. (2011) 91:461–553. 10.1152/physrev.00011.2010 21527731

[13] Aguzzi A Barres BA Bennett ML Microglia: scapegoat, saboteur, or something else? Science (2013) 339:156–61. 10.1126/science.1227901 23307732 PMC4431634

[14] Li Q Barres BA. Microglia and macrophages in brain homeostasis and disease. Nat Rev Immunol. (2018) 18:225–42. 10.1038/nri.2017.125 29151590

[15] Cherry JD Olschowka JA O'banion MK. Neuroinflammation and M2 microglia: the good, the bad, and the inflamed. J Neuroinflamm. (2014) 11:98. 10.1186/1742-2094-11-98 24889886 PMC4060849

[16] Ransohoff RM. A polarizing question: do M1 and M2 microglia exist? Nat Neurosci (2016) 19:987–91. 10.1038/nn.4338 27459405

[17] Benzing WC Wujek JR Ward EK Shaffer D Ashe KH Younkin SG. Evidence for glial-mediated inflammation in aged APP(SW) transgenic mice. Neurobiol Aging (1999) 20:581–9. 10.1016/S0197-4580(99)00065-2 10674423

[18] Bornemann KD Wiederhold KH Pauli C Ermini F Stalder M Schnell L. Abeta-induced inflammatory processes in microglia cells of APP23 transgenic mice. Am J Pathol. (2001) 158:63–73. 10.1016/S0002-9440(10)63945-4 11141480 PMC1850262

[19] Heneka MT Sastre M Dumitrescu-Ozimek L Dewachter I Walter J Klockgether T. Focal glial activation coincides with increased BACE1 activation and precedes amyloid plaque deposition in APP[V717I] transgenic mice. J Neuroinflamm. (2005) 2:22. 10.1186/1742-2094-2-22 16212664 PMC1274341

[20] Schwab C Klegeris A Mcgeer PL. Inflammation in transgenic mouse models of neurodegenerative disorders. Biochim Biophys Acta (2010) 1802:889–902. 10.1016/j.bbadis.2009.10.013 19883753

[21] Bellucci A Westwood AJ Ingram E Casamenti F Goedert M Spillantini MG. Induction of inflammatory mediators and microglial activation in mice transgenic for mutant human P301S tau protein. Am J Pathol. (2004) 165:1643–52. 10.1016/S0002-9440(10)63421-9 15509534 PMC1618683

[22] Yoshiyama Y Higuchi M Zhang B Huang SM Iwata N Saido TC. Synapse loss and microglial activation precede tangles in a P301S tauopathy mouse model. Neuron (2007) 53:337–51. 10.1016/j.neuron.2007.01.010 17270732

[23] Jo S Yarishkin O Hwang YJ Chun YE Park M Woo DH. GABA from reactive astrocytes impairs memory in mouse models of Alzheimer's disease. Nat Med. (2014) 20:886–96. 10.1038/nm.3639 24973918 PMC8385452

[24] Abbas N Bednar I Mix E Marie S Paterson D Ljungberg A. Up-regulation of the inflammatory cytokines IFN-gamma and IL-12 and down-regulation of IL-4 in cerebral cortex regions of APP(SWE) transgenic mice. J Neuroimmunol. (2002) 126:50–7. 10.1016/S0165-5728(02)00050-4 12020956

[25] Patel NS Paris D Mathura V Quadros AN Crawford FC Mullan MJ. Inflammatory cytokine levels correlate with amyloid load in transgenic mouse models of Alzheimer's disease. J Neuroinflamm. (2005) 2:9. 10.1186/1742-2094-2-9 15762998 PMC555557

[26] Vom Berg J Prokop S Miller KR Obst J Kalin RE Lopategui-Cabezas I. Inhibition of IL-12/IL-23 signaling reduces Alzheimer's disease-like pathology and cognitive decline. Nat Med. (2012) 18:1812–9. 10.1038/nm.2965 23178247

[27] Liang X Wang Q Hand T Wu L Breyer RM Montine TJ. Deletion of the prostaglandin E2 EP2 receptor reduces oxidative damage and amyloid burden in a model of Alzheimer's disease. J Neurosci. (2005) 25:10180–7. 10.1523/JNEUROSCI.3591-05.2005 16267225 PMC6725803

[28] He P Zhong Z Lindholm K Berning L Lee W Lemere C. Deletion of tumor necrosis factor death receptor inhibits amyloid beta generation and prevents learning and memory deficits in Alzheimer's mice. J Cell Biol. (2007) 178:829–41. 10.1083/jcb.200705042 17724122 PMC2064547

[29] Irizarry MC Mcnamara M Fedorchak K Hsiao K Hyman BT. APPSw transgenic mice develop age-related A beta deposits and neuropil abnormalities, but no neuronal loss in CA1. J Neuropathol Exp Neurol. (1997) 56:965–73. 9291938 10.1097/00005072-199709000-00002

[30] Irizarry MC Soriano F Mcnamara M Page KJ Schenk D Games D Abeta deposition is associated with neuropil changes, but not with overt neuronal loss in the human amyloid precursor protein V717F (PDAPP) transgenic mouse. J Neurosci. (1997) 17:7053–9. 9278541 10.1523/JNEUROSCI.17-18-07053.1997 PMC6573263

[31] Oddo S Caccamo A Shepherd JD Murphy MP Golde TE Kayed R. Triple-transgenic model of Alzheimer's disease with plaques and tangles: intracellular Abeta and synaptic dysfunction. Neuron (2003) 39:409–21. 10.1016/S0896-6273(03)00434-3 12895417

[32] Wirths O Bayer TA. Neuron loss in transgenic mouse models of Alzheimer's disease. Int J Alzheimers Dis. (2010) 2010:723782. 10.4061/2010/723782 20871861 PMC2943100

[33] Eimer WA Vassar R. Neuron loss in the 5XFAD mouse model of Alzheimer's disease correlates with intraneuronal Abeta42 accumulation and Caspase-3 activation. Mol Neurodegener. (2013) 8:2. 10.1186/1750-1326-8-2 23316765 PMC3552866

[34] Carter SF Scholl M Almkvist O Wall A Engler H Langstrom B. Evidence for astrocytosis in prodromal Alzheimer disease provided by 11C-deuterium-L-deprenyl: a multitracer PET paradigm combining 11C-Pittsburgh compound B and 18F-FDG. J Nucl Med. (2012) 53:37–46. 10.2967/jnumed.110.087031 22213821

[35] Von Bernhardi R Eugenin J. Microglial reactivity to beta-amyloid is modulated by astrocytes and proinflammatory factors. Brain Res. (2004) 1025:186–93. 10.1016/j.brainres.2004.07.084 15464759

[36] Eyupoglu IY Bechmann I Nitsch R. Modification of microglia function protects from lesion-induced neuronal alterations and promotes sprouting in the hippocampus. FASEB J. (2003) 17:1110–1. 10.1096/fj.02-0825fje 12692086

[37] Colangelo V Schurr J Ball MJ Pelaez RP Bazan NG Lukiw WJ. Gene expression profiling of 12633 genes in Alzheimer hippocampal CA1: transcription and neurotrophic factor down-regulation and up-regulation of apoptotic and pro-inflammatory signaling. J Neurosci Res. (2002) 70:462–73. 10.1002/jnr.10351 12391607

[38] Saud K Herrera-Molina R Von Bernhardi R. Pro- and anti-inflammatory cytokines regulate the ERK pathway: implication of the timing for the activation of microglial cells. Neurotox Res. (2005) 8:277–87. 10.1007/BF03033981 16371322

[39] Zhu Y Culmsee C Klumpp S Krieglstein J. Neuroprotection by transforming growth factor-beta1 involves activation of nuclear factor-kappaB through phosphatidylinositol-3-OH kinase/Akt and mitogen-activated protein kinase-extracellular-signal regulated kinase1,2 signaling pathways. Neuroscience (2004) 123:897–906. 10.1016/j.neuroscience.2003.10.037 14751283

[40] Furman JL Sama DM Gant JC Beckett TL Murphy MP Bachstetter AD. Targeting astrocytes ameliorates neurologic changes in a mouse model of Alzheimer's disease. J Neurosci. (2012) 32:16129–40. 10.1523/JNEUROSCI.2323-12.2012 23152597 PMC3506017

[41] Zhang Y Chen K Sloan SA Bennett ML Scholze AR O'keeffe S. An RNA-sequencing transcriptome and splicing database of glia, neurons, and vascular cells of the cerebral cortex. J Neurosci. (2014) 34:11929–47. 10.1523/JNEUROSCI.1860-14.2014 25186741 PMC4152602

[42] Lian H Yang L Cole A Sun L Chiang AC Fowler SW. NFkappaB-activated astroglial release of complement C3 compromises neuronal morphology and function associated with Alzheimer's disease. Neuron (2015) 85:101–15. 10.1016/j.neuron.2014.11.018 25533482 PMC4289109

[43] Lian H Litvinchuk A Chiang AC Aithmitti N Jankowsky JL Zheng H. Astrocyte-microglia cross talk through complement activation modulates amyloid pathology in mouse models of alzheimer's disease. J Neurosci. (2016) 36:577–89. 10.1523/JNEUROSCI.2117-15.2016 26758846 PMC4710776

[44] Bialas AR Presumey J Das A Van Der Poel CE Lapchak PH Mesin L. Microglia-dependent synapse loss in type I interferon-mediated lupus. Nature (2017) 546:539–43. 10.1038/nature22821 28614301

[45] Dissing-Olesen L Ledue JM Rungta RL Hefendehl JK Choi HB Macvicar BA. Activation of neuronal NMDA receptors triggers transient ATP-mediated microglial process outgrowth. J Neurosci. (2014) 34:10511–27. 10.1523/JNEUROSCI.0405-14.2014 25100586 PMC6802598

[46] Davalos D Grutzendler J Yang G Kim JV Zuo Y Jung S. ATP mediates rapid microglial response to local brain injury *in vivo*. Nat Neurosci. (2005) 8:752–8. 10.1038/nn1472 15895084

[47] Paolicelli RC Bolasco G Pagani F Maggi L Scianni M Panzanelli P. Synaptic pruning by microglia is necessary for normal brain development. Science (2011) 333:1456–8. 10.1126/science.1202529 21778362

[48] Schafer DP Lehrman EK Kautzman AG Koyama R Mardinly AR Yamasaki R. Microglia sculpt postnatal neural circuits in an activity and complement-dependent manner. Neuron (2012) 74:691–705. 10.1016/j.neuron.2012.03.026 22632727 PMC3528177

[49] Sipe GO Lowery RL Tremblay ME Kelly EA Lamantia CE Majewska AK. Microglial P2Y12 is necessary for synaptic plasticity in mouse visual cortex. Nat Commun. (2016) 7:10905. 10.1038/ncomms10905 26948129 PMC4786684

[50] Emilsson L Saetre P Balciuniene J Castensson A Cairns N Jazin EE. Increased monoamine oxidase messenger RNA expression levels in frontal cortex of Alzheimer's disease patients. Neurosci Lett. (2002) 326:56–60. 10.1016/S0304-3940(02)00307-5 12052537

[51] Borroni E Bohrmann B Grueninger F Prinssen E Nave S Loetscher H. Sembragiline: a novel, selective monoamine oxidase type b inhibitor for the treatment of alzheimer's disease. J Pharmacol Exp Ther. (2017) 362:413–23. 10.1124/jpet.117.241653 28642233

[52] Deatly AM Haase AT Fewster PH Lewis E Ball MJ. Human herpes virus infections and Alzheimer's disease. Neuropathol Appl Neurobiol. (1990) 16:213–23. 10.1111/j.1365-2990.1990.tb01158.x 2169597

[53] Itabashi S Arai H Matsui T Higuchi S Sasaki H. Herpes simplex virus and risk of Alzheimer's disease. Lancet (1997). 349:1102. 10.1016/S0140-6736(05)62325-2 9107270

[54] Eimer WA Vijaya Kumar DK Navalpur Shanmugam NK Rodriguez AS Mitchell T Washicosky KJ. (2018). Alzheimer's disease-associated beta-amyloid is rapidly seeded by herpesviridae to protect against brain infection. Neuron 99:56–63 e53. 10.1016/j.neuron.2018.06.030 30001512 PMC6075814

[55] Ma KC Nie XJ Hoog A Olsson Y Zhang WW. Reactive astrocytes in viral infections of the human brain express endothelin-like immunoreactivity. J Neurol Sci. (1994) 126:184–92. 10.1016/0022-510X(94)90271-2 7531760

[56] Chen J Yang YF Yang Y Zou P Chen J He Y. AXL promotes Zika virus infection in astrocytes by antagonizing type I interferon signalling. Nat Microbiol. (2018) 3:302–9. 10.1038/s41564-017-0092-4 29379210

[57] Piacentini R Li Puma DD Ripoli C Marcocci ME De Chiara G Garaci E. Herpes Simplex Virus type-1 infection induces synaptic dysfunction in cultured cortical neurons via GSK-3 activation and intraneuronal amyloid-beta protein accumulation. Sci Rep. (2015) 5:15444. 10.1038/srep15444 26487282 PMC4614347

[58] Ortinski PI Dong J Mungenast A Yue C Takano H Watson DJ. Selective induction of astrocytic gliosis generates deficits in neuronal inhibition. Nat Neurosci. (2010) 13:584–91. 10.1038/nn.2535 20418874 PMC3225960

[59] Woo J Im SK Chun H Jung SY Oh SJ Choi N. Functional characterization of resting and adenovirus-induced reactive astrocytes in three-dimensional culture. Exp Neurobiol. (2017) 26:158–67. 10.5607/en.2017.26.3.158 28680301 PMC5491584

[60] Kraft AW Hu X Yoon H Yan P Xiao Q Wang Y. Attenuating astrocyte activation accelerates plaque pathogenesis in APP/PS1 mice. FASEB J. (2013) 27:187–98. 10.1096/fj.12-208660 23038755 PMC3528309

[61] Kamphuis W Kooijman L Orre M Stassen O Pekny M Hol EM. GFAP and vimentin deficiency alters gene expression in astrocytes and microglia in wild-type mice and changes the transcriptional response of reactive glia in mouse model for Alzheimer's disease. Glia (2015) 63:1036–56. 10.1002/glia.22800 25731615

[62] Wilhelmsson U Li L Pekna M Berthold CH Blom S Eliasson C. Absence of glial fibrillary acidic protein and vimentin prevents hypertrophy of astrocytic processes and improves post-traumatic regeneration. J Neurosci. (2004) 24:5016–21. 10.1523/JNEUROSCI.0820-04.2004 15163694 PMC6729371

[63] Sama MA Mathis DM Furman JL Abdul HM Artiushin IA Kraner SD. Interleukin-1beta-dependent signaling between astrocytes and neurons depends critically on astrocytic calcineurin/NFAT activity. J Biol Chem. (2008) 283:21953–64. 10.1074/jbc.M800148200 18541537 PMC2494911

[64] Abdul HM Sama MA Furman JL Mathis DM Beckett TL Weidner AM. Cognitive decline in Alzheimer's disease is associated with selective changes in calcineurin/NFAT signaling. J Neurosci. (2009) 29:12957–69. 10.1523/JNEUROSCI.1064-09.2009 19828810 PMC2782445

[65] Hsiao HY Chen YC Chen HM Tu PH Chern Y. A critical role of astrocyte-mediated nuclear factor-kappaB-dependent inflammation in Huntington's disease. Hum Mol Genet. (2013) 22:1826–42. 10.1093/hmg/ddt036 23372043

[66] Douglass JD Dorfman MD Fasnacht R Shaffer LD Thaler JP. Astrocyte IKKbeta/NF-kappaB signaling is required for diet-induced obesity and hypothalamic inflammation. Mol Metab. (2017) 6:366–73. 10.1016/j.molmet.2017.01.010 28377875 PMC5369266

[67] Zhang Y Reichel JM Han C Zuniga-Hertz JP Cai D. Astrocytic process plasticity and IKKbeta/NF-kappaB in central control of blood glucose, blood pressure, and body weight. Cell Metab. (2017) 25:1091–1102 e1094. 10.1016/j.cmet.2017.04.002 28467927 PMC5576872

[68] Xu Z Xiao N Chen Y Huang H Marshall C Gao J. Deletion of aquaporin-4 in APP/PS1 mice exacerbates brain Abeta accumulation and memory deficits. Mol Neurodegener. (2015) 10:58. 10.1186/s13024-015-0056-1 26526066 PMC4631089

[69] Asraf K Torika N Apte RN Fleisher-Berkovich S. Microglial activation is modulated by captopril: *in vitro* and *in vivo* studies. Front Cell Neurosci. (2018) 12:116. 10.3389/fncel.2018.00116 29765306 PMC5938337

[70] Manocha GD Ghatak A Puig KL Kraner SD Norris CM Combs CK. NFATc2 modulates microglial activation in the abetaPP/PS1 mouse model of alzheimer's disease. J Alzheimers Dis. (2017) 58:775–87. 10.3233/JAD-151203 28505967 PMC6265241

[71] Sieber MW Jaenisch N Brehm M Guenther M Linnartz-Gerlach B Neumann H. Attenuated inflammatory response in triggering receptor expressed on myeloid cells 2 (TREM2) knock-out mice following stroke. PLoS ONE (2013) 8:e52982. 10.1371/journal.pone.0052982 23301011 PMC3536811

[72] Schmid CD Sautkulis LN Danielson PE Cooper J Hasel KW Hilbush BS. Heterogeneous expression of the triggering receptor expressed on myeloid cells-2 on adult murine microglia. J Neurochem. (2002) 83:1309–20. 10.1046/j.1471-4159.2002.01243.x 12472885 PMC2637869

[73] Jones BM Bhattacharjee S Dua P Hill JM Zhao Y Lukiw WJ. Regulating amyloidogenesis through the natural triggering receptor expressed in myeloid/microglial cells 2 (TREM2). Front Cell Neurosci. (2014) 8:94. 10.3389/fncel.2014.00094 24744699 PMC3978349

[74] Grienberger C Rochefort NL Adelsberger H Henning HA Hill DN Reichwald J. Staged decline of neuronal function *in vivo* in an animal model of Alzheimer's disease. Nat Commun. (2012) 3:774. 10.1038/ncomms1783 22491322 PMC3337977

[75] Koffie RM Hashimoto T Tai HC Kay KR Serrano-Pozo A Joyner D. Apolipoprotein E4 effects in Alzheimer's disease are mediated by synaptotoxic oligomeric amyloid-beta. Brain (2012) 135:2155–68. 10.1093/brain/aws127 22637583 PMC3381721

[76] Serrano-Pozo A Mielke ML Muzitansky A Gomez-Isla T Growdon JH Bacskai BJ. Stable size distribution of amyloid plaques over the course of Alzheimer disease. J Neuropathol Exp Neurol. (2012) 71:694–701. 10.1097/NEN.0b013e31825e77de 22805771 PMC3407299

[77] Hosmane S Yang IH Ruffin A Thakor N Venkatesan A. Circular compartmentalized microfluidic platform: Study of axon-glia interactions. Lab Chip (2010) 10:741–7. 10.1039/b918640a 20221562

[78] Taylor AM Blurton-Jones M Rhee SW Cribbs DH Cotman CW Jeon NL. A microfluidic culture platform for CNS axonal injury, regeneration and transport. Nat Methods (2005) 2:599–605. 10.1038/nmeth777 16094385 PMC1558906

[79] Kim S Kim HJ Jeon NL. Biological applications of microfluidic gradient devices. Integr Biol. (2010) 2:584–603. 10.1039/c0ib00055h 20957276

[80] Cho H Hashimoto T Wong E Hori Y Wood LB Zhao L. Microfluidic chemotaxis platform for differentiating the roles of soluble and bound amyloid-beta on microglial accumulation. Sci Rep. (2013) 3:1823. 10.1038/srep01823 23665843 PMC3650586

[81] Togo T Akiyama H Iseki E Kondo H Ikeda K Kato M. Occurrence of T cells in the brain of Alzheimer's disease and other neurological diseases. J Neuroimmunol. (2002) 124:83–92. 10.1016/S0165-5728(01)00496-9 11958825

[82] Subramanian S Ayala P Wadsworth TL Harris CJ Vandenbark AA Quinn JF. CCR6: a biomarker for Alzheimer's-like disease in a triple transgenic mouse model. J Alzheimers Dis. (2010) 22:619–29. 10.3233/JAD-2010-100852 20847401 PMC2988888

[83] Michaud JP Bellavance MA Prefontaine P Rivest S. Real-time in vivo imaging reveals the ability of monocytes to clear vascular amyloid beta. Cell Rep. (2013) 5:646–53. 10.1016/j.celrep.2013.10.010 24210819

[84] Baik SH Cha MY Hyun YM Cho H Hamza B Kim DK. Migration of neutrophils targeting amyloid plaques in Alzheimer's disease mouse model. Neurobiol Aging (2014) 35:1286–92. 10.1016/j.neurobiolaging.2014.01.003 24485508 PMC4248665

[85] Zenaro E Pietronigro E Della Bianca V Piacentino G Marongiu L Budui S. Neutrophils promote Alzheimer's disease-like pathology and cognitive decline via LFA-1 integrin. Nat Med. (2015) 21:880–6. 10.1038/nm.3913 26214837

[86] Park J Wetzel I Baik SH Mook-Jung I Irimia D Cho H The roles of neutrophils in the CNS mediated by reactive microglia in AD. In: Gordon Research Conference: Barriers of the CNS Gordon Research Conference (New London, NH) (2018).

[87] Haenseler W Sansom SN Buchrieser J Newey SE Moore CS Nicholls FJ. A highly efficient human pluripotent stem cell microglia model displays a neuronal-co-culture-specific expression profile and inflammatory response. Stem Cell Rep. (2017) 8:1727–42. 10.1016/j.stemcr.2017.05.017 28591653 PMC5470330

[88] Abud EM Ramirez RN Martinez ES Healy LM Nguyen CHH Newman SA. (2017). iPSC-derived human microglia-like cells to study neurological diseases. Neuron 94:278–293 e279. 10.1016/j.neuron.2017.03.042 28426964 PMC5482419

[89] Park J Wetzel I Marriott I Dreau D D'avanzo C Kim DY. A 3D human triculture system modeling neurodegeneration and neuroinflammation in Alzheimer's disease. Nat Neurosci. (2018) 21:941–95. 10.1038/s41593-018-0175-4 29950669 PMC6800152

[90] Choi SH Kim YH Hebisch M Sliwinski C Lee S D'avanzo C. A three-dimensional human neural cell culture model of Alzheimer's disease. Nature (2014) 515:274–8. 10.1038/nature13800 25307057 PMC4366007

[91] Jiang T Tan L Zhu XC Zhang QQ Cao L Tan MS. Upregulation of TREM2 ameliorates neuropathology and rescues spatial cognitive impairment in a transgenic mouse model of Alzheimer's disease. Neuropsychopharmacology (2014) 39:2949–62. 10.1038/npp.2014.164 25047746 PMC4229581

